# Parkinson's Disease: Is It a Toxic Syndrome?

**DOI:** 10.1155/2010/103094

**Published:** 2010-09-05

**Authors:** Seham A. Gad ELhak, Abdel Aziz A. Ghanem, Hassan AbdelGhaffar, Sahar El Dakroury, Mohamed M. Salama

**Affiliations:** ^1^Toxicology Department, Faculty of Medicine, Mansoura University, Mansoura 35111, Egypt; ^2^Clinical Pathology Department, Faculty of Medicine, Mansoura University, Mansoura 35111, Egypt

## Abstract

Parkinson's disease (PD) is one of the neurodegenerative diseases which we can by certainty identify its pathology, however, this confidence disappeares when talking about the cause. A long history of trials, suggestions, and theories tried linking PD to a specific causation. In this paper, a new suggestion is trying to find its way, could it be toxicology? Can we—in the future—look to PD as an occupational disease, in fact, many clues point to the possible toxic responsibility—either total or partial—in causing this disease. Searching for possible toxic causes for PD would help in designing perfect toxic models in animals.

## 1. Introduction

The term “Parkinson's disease” refers to a neurodegenerative disease that affects several regions of the brain, including the pigmented nuclei in midbrain and brainstem, the olfactory tubercle, the cerebral cortex, and elements of the peripheral nervous system [1].

## 2. Historical Background

Parkinson's disease was first described in a medical context in 1817 by James Parkinson, a general practitioner in London in his *Essay on the Shaking Palsy* [2]. 

 Even earlier many physicians have picked up some of the features of Parkinson's disease and described them in their writings, for example, Franciscus de le Böe (1614–1672) who described tremors and François Boissier de Sauvages de la Croix (1706–1767) who described patients with “running disturbances of the limbs.” 

Wilhelm von Humboldt (1767–1835), the celebrated academic reformer and writer who lived in the era of Parkinson, has described his own neurological condition in a series of letters [3]. 

### 2.1. PD Is a Significant Clinical Problem

PD represents a major clinical problem. Reviewing the multiple clinical findings in PD will show us the need of more researches to reach a possible curative therapy [4].

 A group of neurobehavioral abnormalities can be found in PD such as apathy, fearfulness, anxiety, emotional liability, social withdrawal, increasing dependency, depression, dementia, bradyphrenia, a type of anomia termed the “tip-of-the-tongue phenomenon,” visual-spatial impairment, sleep disturbance, psychosis, and other psychiatric problems [5].

 The variable presentations of PD often cause diagnostic confusion and a delay in treatment. In the early stages, Parkinsonian symptoms are often mistaken for simple arthritis or bursitis, depression, normal aging, Alzheimer's disease, or stroke. Gonera et al. [6] characterized a prodromal phase 4–6 years before the main manifestation in PD patients. During this period, PD patients, compared with normal controls, had a higher frequency of mood disorder, “fibromyalgia,” and shoulder pain. 

 A sum of possible PD manifestations would be *tremors at rest, rigidity, bradykinesia, and loss of postural reflexes* which represents the cardinal manifestations besides possibility of the following.

- Hypomimia (masked facies).
- Speech disturbance (hypokinetic dysarthria).
- Hypophonia.
- Dysphagia.
- Sialorrhea.
- Respiratory difficulties.
- Loss of associated movements.
- Shuffling, short-step gait.
- Festination.
- Freezing.
- Micrographia.
- Difficulty turning in bed.
- Slowness in activities of daily living.
- Stooped posture, kyphosis, and scoliosis.
- Dystonia, myoclonus, orofacial dyskinesia.
- Neuro-ophthalmologic findings.
- Impaired visual contrast sensitivity.
- Visuospatial impairment.
- Impaired upward gaze, convergence, and smooth pursuit.
- Impaired vestibuloocular reflex.
- Hypometric saccades.
- Decreased blink rate.
- Spontaneous and reflex blepharospasm (glabellar or Myerson's sign).
- Lid apraxia (opening or closure).
- Motor findings related to dopaminergic therapy.
- Levodopa-induced dyskinesia (chorea, dystonia, myoclonus, tic) [7].

### 2.2. Neuropathology

Parkinsonism results primarily from abnormalities of basal ganglia function. The basal ganglia include the neostriatum (caudate nucleus and putamen) the external and internal pallidal segments (GPe, GPi), the subthalamic nucleus (STN), and the substantia nigra with its pars reticulata (SNr) and pars compacta (SNc) [1].

They participate in anatomically and functionally segregated loops that involve specific thalamic and cortical areas [8].

Striatum and STN receive glutamatergic afferents from specific areas of the cerebral cortex or thalamus, and transfer the information to the basal ganglia output nuclei, GPi and SNr [1]. 

The striatum also receives prominent dopaminergic input, from the SNc. The nigrostriatal projection terminates predominately at the necks of dendritic spines of the striatal medium spiny output neurons (MSNs). MSN spines also receive corticostriatal terminations. This anatomic arrangement places the dopaminergic inputs in a position to regulate or gate the corticostriatal transmission [9]. 

In Parkinson's disease, the degeneration of dopaminergic SNc neurons and their projections to the striatum is a slowly evolving process that may take decades to develop [10].

 SNc projections to the putamen degenerate earlier than projections to associative or limbic portions of the striatum. Corresponding to this time course of degeneration, the motor symptoms and signs of Parkinson's disease develop before the nonmotor signs. Recognizable motor or nonmotor signs appear only after substantial degeneration of the nigrostriatal neurons (affecting at least 70%), this is due to the remarkable compensatory capacity within the dopaminergic system, or in the circuits it modulates [1].

 The most frequent pathologic substrate for PD is Lewy body disease. The brain is usually grossly normal when viewed from the outer surface. There may be mild frontal atrophy is some cases, but this is variable. 

Histologically, there is neuronal loss in the substantia nigra pars compacta along with compensatory astrocytic and microglial proliferation. Although biochemically there is loss of dopaminergic termini in the striatum, the striatum is histologically unremarkable [11].

 Hyaline cytoplasmic inclusions or Lewy bodies and less well-defined “pale bodies” are found in some of the residual neurons in the substantia nigra [10].

Lewy bodies are proteinaceous neuronal cytoplasmic inclusions [12]. In some regions of the brain, such as the dorsal motor nucleus of the vagus, Lewy bodies tend to form within neuronal processes and are sometimes referred to as intraneuritic Lewy bodies. In most cases, Lewy bodies are accompanied by a variable number of abnormal neuritic profiles, referred to as *Lewy neurites*. 

 Lewy neurites were first described in the hippocampus [13], but are also found in other regions of the brain, including the amygdala, cingulate gyrus, and temporal cortex. 

 At the electron microscopic level, Lewy bodies are composed of densely aggregated filaments [14] and Lewy neurites also are filamentous, but they are usually not as densely packed [13].

Some authors suggested that pre-Parkinsonian manifestations would be nonmotor (e.g., autonomic dysfunction and anosmia) and that the late stage would be associated with cortical Lewy body dementia [15].

### 2.3. Pathogenesis

#### 2.3.1. Δ↽*ψ* *α*-Synuclein: “The Culprit in Parkinson's Disease?” [2]

 Synuclein has gain interest for its role in PD neurodegeneration since the demonstration that *α*-synuclein is the major component of Lewy bodies and Lewy neuritis in idiopathic PD [16].

 The exact mechanisms of how conformational changes of *α*-synuclein induce cell death are currently the subject of intense investigation, but are still poorly understood. It has been suggested that *α*-synuclein oligomers might form pores on intracellular membranes such as the plasma membrane, and may increase cation permeability [16].

*α*-Synuclein has been reported to negatively regulate the activity of tyrosine hydroxylase by maintaining the protein in the dephosphorylated inactive form, thus modulating dopamine biosynthesis in catecholaminergic neurons [17]. 

In addition, *α*-synuclein was proposed to negatively regulate the function of the dopamine transporter in dopaminergic nerve terminals by controlling the amount of the transporter at the plasma membrane [18].

#### 2.3.2. Tau in PD

Tau is a microtubule-binding protein that stabilizes microtubules and promotes their polymerization in neurons. Six isoforms of tau are present in the adult human brain [19].

 While PD is the best known of the *α*-synucleinopathies, tau pathology is also seen in many PD cases. staining of Lewy bodies with multiple tau antibodies has been reported, suggesting cellular colocalization of these two pathologies [20].

#### 2.3.3. Parkin, UCHL1, *α*-Synuclein, and the Ubiquitin-Proteasome Pathway: “Pieces of a Puzzle?” [2]

 Both of the proteins (UCHL1 and parkin) are involved in the ubiquitin-proteasome pathway of abnormal protein degradation which is considered to be a cellular quality control [21].

 When this pathway is impaired, disease can result. Thus, it is possible that mutations in the parkin and UCHL1 genes may lead to malfunction of the pathway [22] and damaged proteins are not degraded. Instead, they form aggregates that ultimately lead to cell degeneration with an unknown mechanism [23].

#### 2.3.4. Mitochondrial Dysfunction in PD

The direct relation between mitochondrial dysfunction and PD came from the postmortem description of complex I deficiency in the substantia nigra of patients with PD. Subsequently, the deficiency was also seen in the skeletal muscle and platelets and there was a decrease in complex I proteins in the substantia nigra of patients with PD [24]. 

 Dopaminergic (DA) neurons are particularly prone to oxidative stress. DA metabolism and auto-oxidation combined with increased iron, decreased total glutathione levels, and mitochondrial complex I inhibition can lead to cell death by exceeding the oxidative capacity of DA-containing cells in the region [25].

#### 2.3.5. Calcium Homeostasis and Excitotoxicity

 It has been an attractive hypothesis for decades that excitotoxicity and disturbance in calcium homeostasis are mechanisms leading to neurodegeneration; disturbed calcium homeostasis is done through the activation of calpains, a family of cysteine proteases [16].

 These are elevated in the mesencephalon of patients with PD but not in other neurodegenerative disorders involving the mesencephalon. *David Park and colleagues* have shown that calpains are activated and required for MPTP-induced neuronal death in mice [26].

### 2.4. Toxic Parkinsonism, Why?

“In one of his Dialogues, the Greek philosopher Plato speculated that our minds contain a block of wax, the size, hardness, and consistency of which varies from person to person. Everything we think or perceive leaves an impression on the wax, and the quality and duration of this impression is the basis of our memory and knowledge.” This metaphor was used by Di Monte [27], to describe the relation between genetics and environmental risk factors in developing PD. 

PD is considered a multifactorial disease resulting from the effect of environmental factors and genetic susceptibility. Linking PD to an environmental cause, however, seems difficult. This is because of the long presymptomatic period [28].

Despite the mysterious cause of PD, the pathologic and neurochemical basis of parkinsonian signs and symptoms have been in part unraveled as discussed earlier. Toxic insults could both modify the structure of *α*-synuclein and interfere with the ubiquitin-proteasomal pathway, thus, promoting *α*-synuclein aggregation and impairing the process of degradation of the abnormal protein [29].

Barlow et al. [30] found that even in utero exposure with subsequent genetic damage can be a risk factor for neurodegenerative diseases, for example, Alzheimer and PD. This can explain the finding of Tsai et al. [31] who described what he has called a young onset parkinsonian disease (YOPD) which is a peculiar group of patients developing PD before 40 years on contrary to normal age group of more than 50 years.

The reason for trying to identify toxic causes of PD (besides future protection) is the search for a perfect PD model. Scientists are trying to unravel the exact cause of PD; also we are trying to find out a perfect therapy and both the therapy and cause need a perfect model to act on. Finding a toxin that can be a true cause of PD would solve the problem.

 So, what are those toxins that can be candidates for toxic PD models? Many have been described including the following.

#### 2.4.1. Pesticides

Many risk factors have been implicated in PD. Since the discovery of MPTP, parkinsonian inducing effects this arouse the possibility of other similar compounds relevant to MPTP, for example, paraquat to induce PD [32].

Numerous classes of pesticides were introduced during the twentieth century. Although PD existed long before the introduction of these pesticides, the thought is that pesticide exposure has contributed to the increased incidence of the disease [33].

**2.4.1.1. Fumigants**

The fumigant class encompasses a variety of agents most commonly used to control insects or fungi in grains, soil, or other various consumables. Fumigants, such as ethylene dibromide, are highly toxic to humans but most adverse actions are nonneuronal [34]. Although there is some evidence that carbon disulfide-based fumigants can induce parkinsonian-like neurotoxicity [35]. This chemical class is not suitable for induction of PD in an animal model.

**2.4.1.2. Fungicides**

Fungicides are agents of a wide variety of chemical structures. *Maneb,* or manganese ethylene bis-dithiocarbamate, is one type with possible parkinsonian symptoms which may be secondary to exposure to the manganese metal core [33].

In fact, maneb is one of the toxins used to induce PD in animal models but usually in combination with other agents specially paraquat [36]. The maneb effect has been undoubtedly documented to increase the severity of PD models which make it one of the good candidates in PD research [37].

**2.4.1.3. Herbicides (Paraquat)**

The possibility that paraquat (1,1_-dimethyl-4,4_-bipyridinium) may damage the nigrostriatal dopaminergic system and therefore contribute to the neuropathology of Parkinson's disease (PD) was first proposed in the mid 1980s following the observation that its chemical structure closely resembles that of MPP^+^(1-methyl-4-phenylpyridinium ion) [38].

Animal studies confirmed the ability of paraquat to induce selective dopaminergic nigrostriatal degeneration [39–41].

Li et al. [40] have developed subacute model for inducing PD in mice through using intraperitoneal injection of paraquat (10 mg/kg) in old C57/bl mice. Similar results were obtained 2 years later by Kuter et al. [41] using a similar approach.

As described earlier, an improvement in modeling of paraquat induced PD was through adding maneb. A combination which improved the results. It also supported the multiple hit theory of PD [42].

The presence of differences between paraquat and MPTP has been questioned and the answer would come from a group of articles in *Toxicological Sciences* journal regarding mechanism of paraquat toxicity. Richardson et al. [43] proposed that paraquat would have different mechanisms for toxicity than MPTP. Later, Ramachandiran et al. [44] have shown that paraquat and MPTP have divergent mechanisms of toxicity. Another hot debate emerged on the same journal regarding the possibility of different mechanisms of neurotoxicity between paraquat and MPTP [45–48].

Despite the debate, there are certainly toxicokinetic and toxicodynamic differences which would give paraquat a unique pattern of neurotoxicity. Despite the fact that paraquat models are still less validated than the previous well-established MPTP models [49], combining maneb with paraquat would introduce a promising modeling technique that can be (in our opinion) a better model specially with the limitation in the other available toxic models.

**2.4.1.4. Insecticides**

There are several subclasses of insecticides, each with their own subdivisions. Many of the compounds in this class are, by design, neurotoxic. Similarities between the insect and human nervous systems can lead to cross-toxicity of these compounds [33].

**2.4.1.4a. Organophosphates**

Although evidence supporting a role for OPs in PD pathogenesis is scant, there are case reports of people with severe Parkinsonism after OP exposure [50]; however, the reversibility of the symptoms and lack of responsiveness to L-dopa are not consistent with PD. A recent family-based case-control study did implicate organophosphates and other insecticides in PD [51].

However, it might be that the effects of OP exposure probably involve disturbance of the balance between the dopamine and acetylcholine systems and not specific pathological changes in the dopamine system which makes them unsuitable in toxic modeling of PD [52].

**2.4.1.4b. Rotenone**

Due to its ability of inhibiting mitochondrial complex I (NADH dehydrogenase), rotenone has become one of the toxic models used to study PD in animals [33, 53].

Chronic systemic exposure to rotenone reproduced many features of PD, including nigrostriatal dopaminergic degeneration and the formation of cytoplasmic inclusions in these neurons. Rats exposed to rotenone showed bradykinesia, postural instability, unsteady gait, and some evidence of tremors [54, 55].

Recently, Cannon et al. [56] reported an improved rotenone model through giving 3 mg/kg daily with observation of behavioral changes including bradykinesia, postural instability/unsteady gait, and rigidity, which occurred maximally after 6 days of treatment where postmortem analysis (immunohistochemistry of the TH+ve neurons in substantia nigra) confirmed the behavioral observations. Others have shown the efficacy of rotenone as a model of PD in mice and suggested its use for assessing candidate anti-Parkinson drugs [57].

Rotenone model can be used as an alternative to other classical PD models, for example, MPTP and 6-OHDA, especially when testing the neuroprotective effects of novel therapeutic modalities [58]. A limiting factor of rotenone models would be the high mortality rate of the examined animals. Another limitation would be the less-accurate results that can be obtained usually in cases of oral administered rotenone [59].

**2.4.1.4c. Organochlorines**

The first clue of a relation between organochlorines and PD was the presence of them in postmortem specimens of PD patients [33]. Kanthasamy et al. [60] have postulated possible mechanisms for the influence of organochlorines on PD patients.

**2.4.1.4c.i. Effects on Dopamine and Dopamine Transporters**

The assumption that organochlorines deplete brain dopamine was supported by the findings of Miller et al. [61]. They measured the expression and activity of the dopamine transporter (DAT) and the vesicular monoamine transporter (VMAT2) in presynaptic terminals of dopaminergic neurons in the striatum. Exposure of C57BL mice to an organochlorine pesticide (increased the expression of both DAT and VMAT2 in the striatum).

Sanchez-Ramos et al. [62] showed that dieldrin is selectively toxic to dopaminergic neurons compared to striatal GABAergic neurons in primary mesencephalic cultures.

**2.4.1.4c.ii. Dieldrin-Induced Oxidative Stress**

Dieldrin exposure was shown to induce ROS production in a number of cell culture models including neuronal cell lines, mouse lung fibroblasts, and in mouse liver [63, 64].

Chun et al. [63] showed that dieldrin exposure induces ROS production in a mouse nigral dopaminergic cell line.

**2.4.1.4c.iii. Dieldrin and Mitochondrial Dysfunction**

Dieldrin exposure has been shown to alter mitochondrial function and induce the release of cytochrome c in a human T cell leukemic cell line [64].

Neuronal death in PD seems to be mitochondrial dependent. Through oxidative mechanisms the soluble pool of cytochrome C in the mitochondrial intermembrane space is increased. For the release of cytochrome C into the cytosol, an activation of Bax to permeabilize the outer mitochondrial membrane appears to be necessary [60].

**2.4.1.4c.iv. Effects on Caspases**

Activation of caspases is an essential step in the apoptotic signaling pathway. Recently caspase 3 has been identified as a critical factor in apoptotic cell death in dopaminergic neurons [65].

PKCd is an important apoptotic substrate for caspase-3, and subsequent studies have implicated caspase-3-mediated proteolytic activation of PKCd in apoptotic cell death [66–69].

Dieldrin treatment induced dose and time-dependent proteolytic cleavage of native PKCd (72 and 74 kDa) into two fragments, 38 kDa regulatory and 41 kDa catalytic subunits, in dopaminergic cells [70, 71].

**2.4.1.4c.v. Effects of Dieldrin on *α*-Synuclein Aggregation and Ubiquitin-Proteasome Function**

Sun et al. [72] findings suggest that dieldrin can alter the function of the ubiquitin-proteasomal degradation machinery to promote protein aggregation.

These encouraging data would increase the organochlorines chance of being a new toxic PD model, especially with their potential of accumulation and so chronic exposure which is one of the characteristics needed to reproduce the exact pathological findings in animal PD models.

**2.4.1.4d. Pyrethroids**

Although pyrethroids are often considered environmentally labile, exposure of mice to the pyrethroid pesticides deltamethrin and permethrin has been demonstrated to increase DAT-mediated dopamine uptake [73–75]. Acute toxicity of pyrethroids is primarily mediated through interaction with sodium channels, leading to prolonged depolarization and hyperexcitation of the nervous system [76].

Pyrethroids have also been shown to be potent releasers of neurotransmitters, including dopamine [73].

Although the mechanism by which pyrethroids are capable of increasing DAT-mediated dopamine uptake is not clear, Elwan et al. [77] suggest that the effects of pyrethroids on DAT are indirect and that longer-term exposures may be capable of damaging cells through an apoptotic mechanism.

The use of pyrethroids in modeling of PD is still far from validation as frequent researches are needed to reach a final exposure protocol.

**2.4.1.5. Metals**

The field of metals in neuroscience has expanded extraordinarily. The biometals, iron, copper, zinc, and manganese participate in many essential activities, and indeed deficiencies can be lethal. Zinc and iron are increased and copper is decreased in the substantia nigra (SN) in Parkinson's disease (PD) [78].

**2.4.1.5a. Iron and PD**

Of great interest is the possible association between iron and nigral melanin as a source of free radicals. Also, iron is postulated to interact with DA or derived catecholic chrome derivatives discussed above, specifically by reacting with hydrogen peroxide that can be generated to produce short-lived hydroxyl radicals [79].

Searches for the mutation of genes associated with iron metabolism have revealed mutations in the ferritin-H, IRP2, and HFE gene in single PD patients. Ceruloplasmin mutations causing protein instability and loss-of-function lead to extrapyramidal symptoms and Parkinsonism, and are characterized by iron accumulation in the substantia nigra [78].

**2.4.1.5b. Manganese: Nontypical PD Manifestations**

Manganese is an essential metal for life, yet chronic exposure to this metal can cause a neurodegenerative disease named manganism. Despite similar clinical pictures, in Parkinson's disease the domaninergic neurons in the substancia nigra are damaged, while in manganism, Mn accumulates in the globus pallidus and striatum, and it damages these two brain structures that control motor function [80]. So despite having some similar manifestations, Mn cannot be ranked as one of the causes of PD.

**2.4.1.5c. Metals and *α*-Synuclein**

Uversky et al. [81] have shown that some metal ions can induce *α*-synuclein fibrillation (*impaired metabolism of normal *α*-synuclein, known to accumulate massively in Lewy bodies (LBs), is considered the primary cause of neurodegeneration in idiopathic PD*). The most effective ions were Al^3^, Fe^3^, Co^3^, Cd^2^, Mn^2^, Cu^2^, Co^2^, in that order. Moreover, they found that combination of metals and pesticides have synergistic effects on *α*-synuclein fibrillation.

The use of metals in toxic animal models however is difficult and did not gain any popularity due to difficulties in application and assessment.

**2.4.1.6. Drug Abuse and PD**

**2.4.1.6a. Amphetamines**

Abuse of amphetamine derivatives is a growing phenomenon in the Western World. Both MA and its derivative, 3,4-methylenedioxymethamphetamine (MDMA, “ecstasy”), are known to be toxic for dopamine nerve terminals, thus replicating striatal DA loss occurring in Parkinsonism. It has been shown that MA and MDMA induce neuronal inclusions in the substantia nigra and corpus striatum of mice [82].

However, in case of drug-induced PD, it is important to interpret neuronal damage—using markers—cautiously as there are usually compensatory changes in these enzymes which may not reflect neurodegeneration [83].

Amphetamine and its derivatives may be applied as toxic models for PD. They are similar to MPTP with safer profile.

**2.4.1.6b. MPTP**

The potent parkinsonian neurotoxin, 1-methyl-4-phenyl-1,2,3,6-tetrahydropyridine (MPTP) causes PD in animals and is one of the most accepted models. This may be due to its capacity to show some of PD pathological findings (SN neuron loss and striatal DA depletion similar to PD) [84].

MPTP-treated animals (including humans) do not exhibit Lewy bodies (LB), a hallmark neuronal inclusion of PD, but old treated primates have inclusions that are somewhat similar. Humans and many other animal species including nonhuman primates, guinea pigs, mice, and cats are susceptible to this neurotoxin [85].

One interesting feature of the MPTP mouse model of PD is the transient nature of the striatal damage in young mice. In contrast, administration of the drug to older mice would result in a permanent loss of nigrostriatal terminals, as well as cell bodies. This recovery potential in young mice allows for modeling of recovery [84].

Various techniques can be used to modulate the action of MPTP to induce the pathological findings needed to reach the desired PD model. One of these techniques is to give probenecid with MPTP to prolong its persistence in the tissues [86].

In sporadic PD, the generation of neural precursor cells in the subependymal zone is impaired by DA depletion, which is the hallmark of PD. MPTP-induced degeneration of dopaminergic SNpc-SVZ fibres impairs NSC proliferation in primates and in mice in a similar fashion to PD [87]. He et al. [88] raised the possibility that migrating neuroblasts in the SVZ may be more vulnerable to MPTP than nigrostriatal dopaminergic neurons in the SN. Furthermore, they suggested that the death and subsequent loss of migrating neuroblasts in the acute or subacute model probably lead to a decreased potential for neurogenesis to some extent.

Several MPTP dosing regimens have been used. The acute regimen consists of multiple systemic administration of MPTP (usually four doses at 2-h intervals per day). The subacute regimen consists of a single systemic administration per day for several consecutive days (usually 5 days) and the chronic regimen through several weeks [89].

The comparison of these different models indicated clearly that different schedules of administration of MPTP mimic distinct stages of the disease and might induce different mechanisms of neuronal death [90].

One of the limitations of MPTP models is their inability to induce behavioral deficits apparent on standard motor mouse tests. However, new techniques for behavioral monitoring have shown promising results, for example, Grid test [84] and its modification, the vertical grid test and modified horizontal grid test [91], swim test [85], or the automated behavioural apparatus, LABORASTM (Laboratory Animal Behaviour Observation, Registration and Analysis System) [92].

Strain differences regarding the response to MPTP has been questioned where C57/bl mice show maximum response compared to BALB/c mice [85, 93, 94]. This does not contradict the old results of Hu et al. [95], who found prominent changes in BALB/c response to MPTP as BALB/c mice still show a possible alternative, though less effective to C57/bl.

An important property of the MPTP-lesioned mouse and nonhuman primate is the potential for intrinsic recovery. This capacity would be of great help in identifying new therapeutic targets for the treatment of PD [96].

## 3. Environment, Genetics, and PD

PD is a multifactorial disease, so despite role of environmental exposure, genetic predisposition in sporadic cases can be broadly grouped into four categories: genes involved in the metabolism of xenobiotics (e.g., CYP2D6, NAT2, GSTs), neurodegeneration (e.g., NOS), the functioning of dopaminergic neurons (e.g., dopamine transporters and receptors), and linkage-derived genes (e.g., UCHL1, alpha-synuclein) [97].

There is increasing evidence that genes involved in inherited forms of the disease may act as predisposing factors in the sporadic forms of PD. So, new models of PD produced in vivo on transgenic animals and in vitro on transfected cell cultures are considered important because they may be helpful in discovering the pathophysiology of PD [98]. 

Meredith et al. [89] have classified genetic models into three categories. First, mouse models based on the deletion of genes important for the development or maintenance of DA neurons or their phenotypes. Second, mouse or rat models based on expression or deletion of genes known to cause familial forms of PD. Finally, a third class of genetic models is based on virally mediated expression of genes or mutations known to cause familial PD, usually in nigrostriatal DA neurons.

 Despite having some features of the human disease, genetic models do not provide an ideal choice for assessing regenerating capacity of new therapeutic approaches and so are of less use for pharmaceutical development [99].

## 4. Conclusion

In our search for an ideal toxic model of PD, we could not find a perfect one. It seems that all toxins when used alone can precipitate only some characters of PD pathology but not the exact PD condition. 

These same findings may be the cause of the Manning-Bog and Langston [100] advice of using *model fusion* to overcome the single model limitation through combining genetic with toxic models. We would encourage the use of toxins combination in PD modeling as a new type of model fusion.
